# Editorial: Developmental Programming of Metabolic Diseases

**DOI:** 10.3389/fendo.2021.781361

**Published:** 2021-10-21

**Authors:** Sarah J. Glastras, Damaskini Valvi, Amita Bansal

**Affiliations:** ^1^Department of Diabetes, Metabolism and Endocrinology, and Kolling Institute, Royal North Shore Hospital, Sydney, NSW, Australia; ^2^Sydney Medical School, University of Sydney, NSW, Australia; ^3^Department of Environmental Medicine and Public Health, Icahn School of Medicine at Mount Sinai, New York, NY, United States; ^4^Australian National University (ANU) Medical School, and John Curtin School of Medical Research, College of Health and Medicine, Australian National University, Canberra, ACT, Australia

**Keywords:** developmental programming, metabolism, fructose, vitamin B12, mitochondrial dysfunction, adiposity, metformin, adipocyte precursors

Environmental exposures from pre-conception through early postnatal life are associated with adverse consequences for the offspring and may increase the likelihood of developing chronic diseases in adult life, such as type 2 diabetes, obesity, cardiovascular disease, and chronic kidney disease (1–5). In this Research Topic series involving 8 peer-reviewed articles, we focus on maternal factors that can lead to adverse developmental programming of offspring metabolism (Smith et al.; Damron et al.; Behere et al.), consider cellular mechanisms of foetal programming (Pendleton et al.; Ying and Simmons; Poon), discuss the programming effects on various organs specifically liver, skeletal muscle, adipose tissue and the brain (Pendleton et al.; Ying and Simmons; Poon), and draw attention to intervention strategies that may prevent the risk of metabolic disturbance to the offspring (Li et al.; 6).

Maternal exposure to either nutrient excess or deficit can have long-term consequences for offspring’s metabolic health. The study by Smith et al. using a novel guinea pig model of maternal high-fructose diet exposure, demonstrates perturbed metabolic outcomes for the offspring, including higher free fatty acids, increased serum uric acid and triglycerides (Smith et al.). In addition Damron et al. observed that maternal high-fat feeding from 4 weeks prior to mating throughout pregnancy in C57BL/6J mice induced sex-specific effects in the offspring, such that male offspring had increased adiposity, impaired glucose tolerance and reduced pancreatic insulin secretion (Damron et al.). Similar metabolic perturbations have been previously described in the offspring whose mothers were exposed to high-fat diet or environmental chemicals during pregnancy (7–10). Furthermore, Behere et al, in a systematic review of studies conducted in India, observed high prevalence of vitamin B12 deficiency in women in India during pregnancy with potential adverse outcomes for the offspring’s metabolic health (Behere et al.). Their systematic review supports the inclusion of vitamin B12 to existing nutritional programs in India for extended benefits on outcomes in pregnancy and offspring health besides control of anaemia. Together these studies indicate that maternal exposure to a sub-optimal environment can impair offspring’s metabolic health in the long-term and highlight a need for interventions around pregnancy, which will prove beneficial in curtailing these long-term metabolic sequelae.

The mechanisms underpinning the association of maternal factors with increased susceptibility of the offspring to develop obesity, type 2 diabetes, heart disease and other components of metabolic syndrome in adulthood are not fully elucidated and increasingly investigated. Inflammation, oxidative stress, mitochondrial biogenesis, lipid metabolism and placental function are regarded as key regulators of foetal programming (11–13). Pendleton et al. reviewed the evidence that intrauterine growth restriction induces placental insufficiency, which has profound effects on the developing foetus, with metabolic dysfunction seen in skeletal muscle and liver (Pendleton et al.). Similar metabolic dysfunction following intrauterine growth restriction has been described previously in pancreatic islets also (14, 15). Specifically, these are linked to 10-maladaptive mitochondrial changes leading to impaired mitophagy (Pendleton et al.). Mitochondrial dysfunction is implicated in a wide variety of chronic metabolic diseases including but not limited to diabetic kidney disease, obesity and type 2 diabetes (13, 16). Simmons and Ying reviewed the latest evidence from experimental models about adipocyte precursors lineage and regulation mechanisms during development and in adulthood. Their review summarize important differences in adipogenesis mechanism during development compared to adult adipogenesis and underscores the need for future investigation on developmental adipocyte precursors in humans and their role in the developmental origins of health and disease (Ying and Simmons). In the review by Poon, the evidence of the impact of maternal obesity on hypothalamic neurocircuitry highlights that prenatal high-fat diet exposure increases neurogenesis of neuropeptide systems in offspring brain and influences behaviour with respect to food seeking behaviour (Poon). The review specifically discussed the role of inflammatory mediators, chemokines, in this process, illustrating how upregulation of chemokines following a high fat diet intake impacts brain neurochemistry in offspring, thereby mediating the neurogenic effects of a high fat diet on hypothalamic peptide neurons (Poon). Taken together, we are gaining greater understanding of the mechanisms underpinning developmental programming of metabolic disease and reputed players, such as the mitochondria and immune system are emerging to play a central role.

Preventing adverse programming at preconception is perhaps the most attractive approach that promotes metabolic health starting early in life. In this Research Topic series, Xu et al. discuss the potential role of metformin therapy during gestation, and Li et al. investigate the role of low dose aspirin to prevent kidney damage in LPS-induced preeclampsia, discovering that aspirin inhibits the WNT5A and NF-kB signalling pathway. In the context of maternal overnutrition, low dose hydralazine, a demethylating agent safe in pregnancy has also demonstrated reduced renal fibrosis in rodent offspring (17, 18). Ongoing future research to establish safe and effective treatments that can be safely administered before or during pregnancy are urgently needed to prevent adverse developmental programming of the foetus and improve the long-term health of the offspring. Given our knowledge that postnatal high-fat diet feeding significantly amplifies the adverse metabolic effect of maternal obesity (19), pharmacological strategies to mitigate neurological and psychological drive towards food seeking behaviour are needed. In the past decade, there have been significant advances in pharmacotherapy for weight reduction which include glucose-like peptide-1 (GLP-1) receptor agonists, utilised previously in the offspring of a mouse model of maternal obesity to rescue offspring metabolic dysfunction (20). More recently, tesofensine, a novel monoamine reuptake inhibitor that inhibits both norepinephrine, serotonin, and dopamine reuptake function, has been developed as a promising new treatment for obesity (21). There remains a paucity of evidence as to the benefit to mother and the offspring of maternal weight reduction prior to conception in females with obesity.

Though genetic predisposition and postnatal environmental exposures are important aetiological factors for the development of chronic disease over the life-course, maternal health and *in utero* environmental exposures can perturbate metabolic programming in the foetus through direct mechanisms with long-lasting adverse metabolic effects over the lifespan. The preconception and pregnancy windows offer unique opportunities for interventions that promote optimal health for both the mother and the offspring. Findings summarized in this Special Issue provide novel insights into the mechanisms underlying the developmental origins of metabolic diseases and highlight treatments that prevent metabolic risks starting early in life. These findings will inform the design of more effective interventions focused on the prevention of environmental triggers, maternal nutrition and/or potential therapeutic targets.

## Author Contributions

SG, DV, AB conceptualized, wrote, edited, and approved the final version of the manuscript. All authors contributed to the article and approved the submitted version.

## Conflict of Interest

The authors declare that the research was conducted in the absence of any commercial or financial relationships that could be construed as a potential conflict of interest.

## Publisher’s Note

All claims expressed in this article are solely those of the authors and do not necessarily represent those of their affiliated organizations, or those of the publisher, the editors and the reviewers. Any product that may be evaluated in this article, or claim that may be made by its manufacturer, is not guaranteed or endorsed by the publisher.
